# FCN-PD: An Advanced Deep Learning Framework for Parkinson’s Disease Diagnosis Using MRI Data

**DOI:** 10.3390/diagnostics15080992

**Published:** 2025-04-14

**Authors:** Manal Alrawis, Farah Mohammad, Saad Al-Ahmadi, Jalal Al-Muhtadi

**Affiliations:** 1 Center of Excellence and Information Assurance (CoEIA), King Saud University, Riyadh 11543, Saudi Arabia; 441204102@student.ksu.edu.sa (M.A.); salahmadi@ksu.edu.sa (S.A.-A.); jalal@ksu.edu.sa (J.A.-M.); 2 Department of Computer Science, and Technology, Arab East Colleges, Riyadh 11583, Saudi Arabia; 3 College of Computer & Information Sciences, King Saud University, Riyadh 11543, Saudi Arabia

**Keywords:** Parkinson’s disease, U-Net, EfficentNet, attention mechanism, FCN

## Abstract

**Background/Objectives:** Parkinson’s disease (PD) is a progressive neurodegenerative disorder characterized by motor dysfunction, cognitive decline, and a diminished quality of life. Early and accurate diagnosis is essential for effective disease management. However, traditional diagnostic approaches, which rely on clinical observations and subjective assessments, often lead to delays and inaccuracies. This research aims to address these limitations by proposing FCN-PD, an advanced deep learning framework for accurate PD diagnosis using MRI data. **Methods:** The FCN-PD framework incorporates a hybrid feature extraction phase that combines EfficientNet to capture local spatial details and attention mechanisms to extract global contextual information. These features are then processed by a Fully Connected Network (FCN) for final classification. This architecture enables the model to effectively represent hierarchical features and handle high-dimensional MRI data while mitigating issues such as overfitting and feature redundancy. **Results:** The performance of FCN-PD was evaluated on three publicly available MRI datasets. On the PPMI dataset, it achieved an accuracy of 97.2%, outperforming traditional CNN-based models by 5.3%. On the OASIS dataset, the model achieved 95.6% accuracy, and on the MIRIAD dataset, it reached 96.8% accuracy. These results establish FCN-PD as a superior alternative to existing PD diagnostic methods. **Conclusions:** FCN-PD demonstrates significant improvements in diagnostic accuracy and efficiency for Parkinson’s disease using MRI data. Its robust architecture effectively captures both local and global features, making it a promising tool for clinical integration and early PD detection, ultimately contributing to better patient outcomes.

## 1. Introduction

Parkinson’s disease (PD) is a progressive neurological condition that mainly impacts motor function [1]. It develops as a result of damage to or loss of nerve cells in the brain, especially those that produce dopamine [2]. Dopamine is a chemical messenger that plays a key role in transmitting signals to the region of the brain responsible for regulating muscle coordination [3]. As dopamine levels decrease, the symptoms of PD begin to manifest. The disease is progressive, meaning it worsens over time, and it can significantly impact an individual’s ability to perform daily activities. The most common symptoms of Parkinson’s disease, as already mentioned in Table 1, include tremors (shaking), muscle rigidity, bradykinesia (slowness of movement), and postural instability, which may lead to balance problems and falls. People with PD may also experience non-motor symptoms, such as sleep disturbances, depression, memory problems, and changes in speech or handwriting [4]. Research suggests that exposure to certain toxins, traumatic brain injury, or a history of viral infections may increase the risk of developing the disease. However, the complex nature of the disease means there is still much to be explored in understanding its precise causes [5].

Parkinson’s disease progresses in stages, typically categorized into five phases, known as the Hoehn and Yahr scale [6]. In Stage 1, symptoms are mild and usually affect only one side of the body, with slight tremors or rigidity. In Stage 2, symptoms worsen, impacting both sides of the body, causing difficulties with walking and balance, but individuals can still live independently. Stage 3 marks a significant decline in balance and motor coordination, with falls becoming more common, and daily tasks become challenging. Stage 4 brings severe disability; individuals require assistance with daily activities, though they may still be able to walk with help. Finally, Stage 5 is the most advanced stage, where individuals are often wheelchair-bound or bedridden, with a high level of dependency on caregivers [7]. There is no definitive test for Parkinson’s, so doctors typically diagnose it based on medical history, symptoms, and physical and neurological examinations. Imaging tests, like MRI or CT scans, may be used to rule out other conditions but cannot definitively diagnose Parkinson’s. In some cases, a DaTscan, which uses a special imaging technique to assess dopamine levels in the brain, can help confirm the diagnosis [8]. Additionally, a positive response to Parkinson’s medications, like levodopa, may further support the diagnosis; most individuals find relief from their symptoms when using these medications.

Conventional ways of diagnosing Parkinson’s disease have some shortcomings as they mainly depend on assessments and neurological exams which may not detect the disease in its stages when symptoms are not yet prominent enough to indicate significant brain damage [9]. Moreover, these methods do not offer a test for distinguishing Parkinson’s from disorders that share similar symptoms. Imaging methods such as MRI or CT scans are useful for ruling out conditions, though they may not be effective in detecting the initial phases of PD [10]. Additionally, the assessment of symptoms can vary due to its nature in the early stages when symptoms are mild and less obvious. Moreover, the lack of a biomarker for PD often leads to diagnoses relying heavily on judgment, which can result in errors or delays in identifying the condition.

Machine learning and deep learning technologies provide benefits compared to diagnostic approaches for PD. By examining datasets like records and information, wearable devices or studying speech patterns can help detect subtle signs that may point towards the initial phases of PD [11]. In contrast to approaches that depend on physical indications typically seen in later phases of the illness, machine learning has the ability to identify initial signs of the condition before significant symptoms emerge allows for earlier intervention and tailored treatment strategies. Conversely, deep learning, a form of machine learning, demonstrates proficiency in analyzing datasets such, as medical imagery (MRI scans) CT scans, PET scans) and recordings of speech. Deep learning models have the ability to identify details in brain scans or voice features that might be missed by professionals to enhance precision and minimize diagnostic mistakes. Moreover, digital approaches allow for monitoring using devices, a feature not feasible with conventional techniques, enabling quicker and more up-to-date observations on disease development [12]. Therefore, machine learning and deep learning present accurate methods for detecting and handling Parkinson’s disease in its early stages compared to standard clinical assessments.

The FCN-PD framework suggested takes an approach to guarantee the diagnosis of PD from MRI data by following a structured process. It starts with gathering data by obtaining high quality MRI scans to create the dataset. During this, the stage U net [13] is used for segmentation of the brain regions, identifying areas like the substantia nigra. Meanwhile, Auto Encoders [14] are utilized to eliminate noise and improve feature quality by preserving elements. In the stage of the process, EfficentNet [15] is utilized to gather spatial information, like cortical thickness and texture changes, while attention mechanisms are employed to analyze overall connections ensuring a complete grasp of both the structural and contextual patterns in the brain. Lastly, during the final phase, the FCN [16] carries out classification by combining local and global features through its innovative shifted window attention mechanism.

### Research Contribution

The eye-catching contributions of the proposed work are as follows:

- The key contribution of this work is the introduction of a hybrid EfficentNet and attention mechanism that capture both local and global MRI features, enhancing precision in identifying PD-related anomalies.
- Utilized the FCN for efficient, context-aware classification, achieving superior accuracy and scalability for high-resolution MRI data.
- Enhanced diagnostic accuracy and interpretability by integrating multi-scale features with normalized linear transformation, ensuring reliable and clinically relevant outcomes.

The rest of this paper is structured as follows: Section 2 offers a comprehensive review of the literature, while Section 3 details the core methodology. Section 4 presents the experimental results and evaluation, and finally, Section 5 concludes the paper and outlines directions for future work.

## 2. Literature Review

Several studies, as highlighted in Table 2, have investigated the potential use of MRI and speech signal-based systems for diagnosing Parkinson’s disease. Shah [16] in their research paper presented a tool utilizing CNN with the goal of distinguishing individuals with PD from those without the condition (HC). They assessed the systems effectiveness through measurements such as accuracy rate and ability to detect positives and negatives indicated by the area under the curve (AUC). The outcomes revealed an enhancement of 3.9% in all performance indicators compared to existing approaches described in studies. In an investigation, Lamba [17] examined speech data collected from the University of California Irvine’s machine learning repository to assess how well models handle datasets and found that using SMOTE for dataset balancing helped address this issue effectively. They discovered that combining a genetic algorithm with a random forest classifier led to better outcomes with an accuracy rate of 95.58%, surpassing previous approaches in the same domain.

In a study by Loh [18], they presented a learning model using EEG data to automate the classification of PD. The research involved examining EEG recordings from 16 individuals without PD and 15 PD patients to assign them into three groups; individuals with no PD diagnosis; PD patients taking medication; and PD patients not taking medication, as outlined in the study by Wang et al. In a study on detecting PD by stage [19] researchers utilized a sophisticated deep learning algorithm that included factors like REM sleep problems and issues with the sense of smell as well as data on cerebrospinal fluid biomarkers and dopaminergic imaging results in their model. By analyzing information from 183 individuals and 401 patients with staged PD, their method outperformed twelve other machine learning models and ensemble techniques impressively with an accuracy rate of 96.45%. The research also emphasized the significance of characteristics in the process by employing Boosting methods to determine their impact.

The methods proposed by Aljalal [20] utilize the Common Spatial Pattern (CSP) to identify PD in two states: off medication and on medication. EEG signals were processed through artifact removal. Feature extraction procedures were used that involve assessing variance, band power, energy, and entropy. Subsequently, various algorithms such as forest, support vector machine and k nearest neighbor were employed for classification purposes. Tested on datasets from San Diego and UNM universities, the CSP combined with log energy entropy yielded promising outcomes with accuracy rates reaching around 99% for cases without medication and 95% to 98% for those with medication. These approaches also pinpointed alpha and beta frequency band characteristics as crucial elements influencing the model’s performance, highlighting their value in settings. Quan et al. [21] examined both fixed and changing speech traits when diagnosing PD, focusing on differences in articulation shifts between individuals and those affected by PD. Their study pointed out variations in how sounds change and patterns in the pitch curve over time. Testing using a method called 10 cross validation showed that this new way performed better than fashioned models that use fixed characteristics significantly improving how accurately things are detected.

Wong [22] presented the Deep Multi Variate Vocal Data Analysis (DMVDA) framework in their research work. This framework employed an algorithm that was specifically crafted to handle speech features and incorporated techniques for sampling acoustic data. This allowed the system to effectively examine a spectrum of datasets. The DMVD approach showcased a 3% enhancement in performance compared to methods for identifying symptoms of PD. This underscores its promise in scrutinizing diverse datasets.

**Table 2:** Comparative analysis of existing PD detection models.

Ref.	Core Methodology	Accuracy	Limitations
[23]	FLIRT image registration and BET non-brain tissue scraper	0.9620 (Accuracy), 0.9452 (F1 score), 0.9407 (Precision), 0.9536 (Recall), 3D CNN R = 0.9150, R^2^ = 0.8372 (Severity)	Performance may degrade with noisy or low-quality input data.
[24]	Hybrid method: data augmentation, pretrained CNN (VGG16), feature selection via Binary Grey Wolf Optimization (BGWO), classification with SVM	99.8% accuracy	Relies on a specific handwriting dataset, limited generalization to other types of data.
[25]	Two-stage diagnostic system: L1 regularized SVM for feature refinement, classification via deep neural network	100% (LOSO cross-validation), 97.5% (k-fold cross-validation)	Relies on specific datasets, may not generalize to all PD populations.
[26]	Deep learning with Harris Hawks Optimization (HHO), model comparison with AlexNet, GoogleNet, MobileNetV2, ResNet18, ResNet50	94.12% accuracy, 100% accuracy in model averaging	Focus on handwritten data, which may not cover all PD detection methods.
[27]	Hybrid model with dataset balancing using three sampling techniques (Random Oversampling, SMOTE), evaluated using	97% recall, 99% AUC	Dataset imbalanced issue addressed; Generalizability to larger datasets may be a concern.
[28]	(XGBoost, LightGBM, Bagging, AdaBoost, Support Vector Machine)	96% accuracy, 96% AUC, 100% sensitivity, 94.43% specificity with LightGBM	Relies on vocal features; Other clinical features may not be included.

From the above discussion, it has been concluded that accurate and early diagnosis of PD is vital for effective treatment, yet traditional diagnostic methods, relying on clinical observations and subjective assessments, often result in delays and inaccuracies. While computer-based approaches using traditional machine learning and basic deep learning models have shown potential in enhancing diagnostic precision, they are often limited by their inability to seamlessly integrate local and global features, process high-resolution MRI data efficiently, and avoid overfitting. The proposed FCN-PD framework overcomes these challenges through a systematic pipeline designed for reliable PD diagnosis using MRI data. It starts with high-quality MRI scans, which undergo preprocessing via U-Net for precise segmentation of critical brain regions, such as the substantia nigra, and Auto Encoders to eliminate noise while preserving essential structural details. In the feature extraction phase, EfficentNet captures localized spatial features, such as cortical thickness and texture variations, while attention mechanisms ensure a deeper understanding of global structural dependencies. The final diagnosis phase employs the FCN, which integrates these local and global features using a shifted window attention mechanism, enabling hierarchical classification and efficient processing of high-resolution data.

## 3. Proposed Methodology

**Figure 1:**
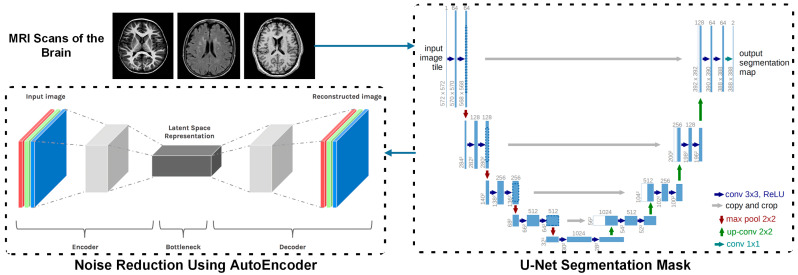
The architecture of FCN-PD for PD diagnosis (noise Reduction and segmentation).

**Figure 2:**
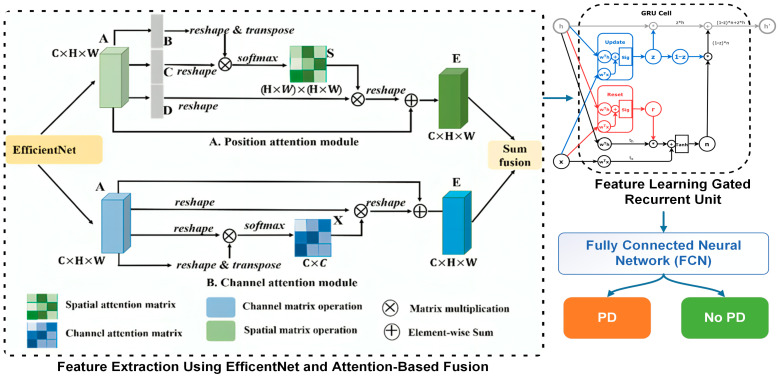
The architecture of FCN-PD for PD diagnosis (feature extraction and classification).

This section discusses the core methodology of the FCN-PD. The key steps of the proposed work as depicted in Figure 1 and Figure 2 are: data collection, preprocessing, data augmentation feature extraction, feature learning, and final diagnosis. A detailed description of each step is presented in the subsections below.

### 3.1. Data Collection

The datasets used in this study were carefully selected to ensure the inclusion of high-quality and diverse MRI scans for the detection of PD. To achieve robust model performance, three publicly available datasets as presented in Table 3 were utilized: The Parkinson’s Progression Marking Project (PPMI), the Open Access Series of Imaging Studies (OASIS-3), and the MRI and Dementia Dataset (MIRIAD). These datasets provide a combination of PD and healthy control cases, offering a wide range of imaging data that captures structural variations in the brain.

The PPMI is a dataset that researchers can access over time to find markers for PD. This database combines MRI scans with information and genetic data to offer an understanding of how the disease develops over time. In total, there are 2500 scans from than 1000 participants in the MRI dataset. These participants include both individuals with PD (1500 scans) and those who are healthy (1000 scans). Typically, each participant gets scans performed over years which allows scientists to examine the progression of the disease closely. For instance, structural MRIs using T’ weighted are processed in advance with techniques such as FLIRT and BET to guarantee top notch data for analysis.

The OASIS dataset is a part of the Open Access Series of Imaging Studies that focuses on brain imaging in relation to aging and neurodegenerative conditions like PD. While its main purpose is to study Alzheimer’s disease, it also contains MRI scans of PD patients and individuals without health issues. This dataset consists of 1200 MRI scans. Around 200 are from PD patients and the remaining 1000 are from individuals. The information provided includes T1-weighted MRIs along with metadata such as participant age, cognitive function status, and medical background for each scan. The MIRAD dataset is a collection of neuroimages that concentrates on dementia and Parkinson’s disease research. Subjects’ high-quality MRI scans are included in this collection from a group of 63 individuals; 32 scans are from Parkinson patients and 31 are from healthy individuals. The dataset mainly comprises T1-weighted MRIs that have been processed to eliminate artifacts and improve image clarity. This resource is especially beneficial for investigating early-stage Parkinson’s disease as it includes participants with mild symptoms.

### 3.2. Preprocessing

In the data preprocessing step for MRI-based PD diagnosis, the integration of U-Net and Auto Encoders provides a comprehensive solution that enhances the quality and usability of the data for subsequent deep learning analysis. These models are selected for their ability to address critical challenges, such as precise brain region segmentation, noise reduction, and normalization, which are fundamental for accurate diagnosis. Algorithm 1 shows the preprocessing of the MRI data.
**Algorithm 1:** U-Net- and Auto Encoder-based preprocessing for MRI data**Require:**$\;\mathrm{MRI}\;\mathrm{dataset}\;D\;=\;{\left\{x_{i}\right\}}_{i=1}^{N},$ U-Net model U, AutoEncoder model A **Ensure:** Preprocessed dataset $D_{preprocessed}$
1.Initialize $D_{preprocessed}$ = ∅2.**for** each MRI image $x_{i}\;\in \;D$ **do**3.**Segmentation Step:** Apply U-Net U on $x_{i}$ to generate segmentation mask $M_{i}$: $M_{i}=U(x_{i})$
4.Extract segmented region $x_{i,seg}=x_{i}⊙M_{i}$, where ⊙ denotes element-wise multiplication.5.**Noise Reduction Step:** Pass segmented image $X_{i,seg}$ through AutoEncoder A to obtain denoised image c: ${\hat{X}}_{i}=A(X_{i,seg})$
6.**Validation Step:**7.**if** Reconstruction error ${L=||X_{i,seg}-{\hat{X}}_{i}\;||}^{2}$ is below threshold ∈ **then** Add ${\hat{X}}_{i}$ to $D_{preprocessed}$8.**Else**9.Flag $X_{i}$, for manual review.10.**end if**11.**end for**12.**return** $D_{preprocessed}$

The U-Net model, specifically designed for biomedical image segmentation, plays a pivotal role in isolating the brain regions most relevant to PD, such as the substantia nigra [29]. This model operates through a contracting path (encoder) to capture contextual information and an expanding path (decoder) for precise localization. Mathematically, the U-Net applies a convolutional kernel $K\in R^{k\times k}$ to the input image $X\in R^{H\times W\times D}$, producing feature maps F as:

(1) F=ReLU(X∗K+b)

where b is the bias, ∗ represents the convolution operation, and ReLU (Rectified Linear Unit) introduces non-linearity. The skip connections between corresponding layers in the encoder and decoder ensure the retention of fine-grained spatial information, crucial for segmenting small yet significant regions. By performing pixel-wise classification, U-Net generates a binary mask $M\in {\left\{0,1\right\}}^{H\times W\times D}$, where:

(2) $$M_{i,J,K}=\left\{\begin{array}{l} 1,\;\;if\;voxel\;belongs\;to\;the\;target\;region\\ 0,\;\;Otherwise \end{array}\right.$$

This precise segmentation allows the model to focus on the structural changes in the brain that are characteristic of PD, ensuring that irrelevant regions do not introduce noise or ambiguity into the analysis. Parallelly, Auto Encoders are employed to address noise reduction and normalization in MRI images. An Auto Encoder is an unsupervised learning model that compresses the input X into a latent representation $z\in R^{d}$ through an encoder function $f_{\theta }:X\to z$ and then reconstructs it using a decoder function $g_{\phi }:z\to X^{\prime }g$. The model minimizes the reconstruction loss as:

(3) $$L={\left||X-X^{\prime }|\right|}^{2}$$

where $X^{\prime }=g_{\phi }(f_{\theta }(X))$ is the reconstructed image. This process filters out irrelevant artifacts while retaining essential structural information. Variations in imaging conditions, such as scanner noise or patient movement, can introduce inconsistencies in the dataset. The Auto Encoder effectively handles these issues by learning the underlying patterns of the MRI images, ensuring uniformity and consistency in the data. Additionally, the latent space z captures the most critical features of the input, which can later be used to enhance the performance of deep learning models in the diagnosis phase.

Together, these models address the unique requirements of preprocessing MRI data for PD diagnosis [30]. U-Net ensures that the analysis is focused on the most relevant brain regions, eliminating the interference of unrelated structures, while Auto Encoders enhance the data quality by reducing noise and normalizing variations. This dual approach not only prepares the dataset for efficient feature extraction but also significantly improves the robustness and accuracy of the downstream deep learning model. The combination of segmentation and denoising ensures that the inputs to the diagnostic model are both precise and high-quality, providing a strong foundation for achieving reliable and early detection of PD.

Following the segmentation and noise reduction steps in the preprocessing pipeline, MRI images have been denoised and validated to create the preprocessed dataset $D_{preprocessed}$. The next and most important phase is data augmentation, which addresses the scarcity of MRI data by enabling the generation of realistic synthetic images that enhance dataset diversity. This reduces overfitting, improves model generalization, and enhances the accuracy of Parkinson’s disease diagnosis. In this work, data augmentation is performed using Generative Adversarial Networks (GANs), which consist of a generator G and a discriminator D, trained in an adversarial manner. The generator G(z), where z is random noise sampled from a normal distribution $z∼N\left(0,\;1\right)$, learns to produce synthetic MRI images. The discriminator evaluates whether the provided images are real from $D_{preprocessed}$ or fake (generated by G). The adversarial training optimizes the following loss function:

(4) .DminEx∼pdataxDmaxlogDx+Ez∼pzzlog1−DGz

The process begins by initializing the GAN architecture. The generator employs transposed convolutional layers to up sample the noise and generate MRI images, while the discriminator uses convolutional layers to classify images as real or fake. The training alternates between the generator and the discriminator, with both networks optimized using the Adam optimizer. The learning rate is tuned for stable convergence, ensuring the generator produces increasingly realistic MRI images over successive iterations

### 3.3. Feature Extraction

Feature extraction is a critical step in MRI-based PD diagnosis, as it translates complex, high-dimensional raw data into meaningful and compact representations that capture the underlying patterns and structures relevant to the disease [31]. By focusing on specific features such as cortical thickness, texture, or volumetric data, feature extraction ensures that the subsequent analysis is efficient and precise, reducing noise and irrelevant information. In the proposed methodology, the typical CNN architecture that is EfficentNet has been used for spatial and local feature extraction and attention-based fusion for integrating global dependencies provides a powerful and comprehensive approach to handle the diverse characteristics of MRI data [32]. Algorithm 2 shows the working flow of this process.
**Algorithm 2:** Feature extraction using EfficentNet and Attention-Based FusionRequire: MRI dataset mathematically ${D=\{x_{i}\}}_{i=1}^{N}$, EfficentNet model R, Attention module A Ensure: Extracted hybrid features $F_{Hybrid}$1.Initialize $F_{Hybrid}$ = ∅
2.for each MRI image $x_{i}\;\in \;D$ do3.Step 1: Local Feature Extraction Pass $X_{i}$ through EfficentNet R to extract local features: $F_{FFNet}=R{(X}_{i})$4.Flatten $F_{FFNet}$ into tokens T = ${\{T}_{1},T_{2},\ldots T_{n}\}$.5.Step 2: Global Context via Attention Compute attention scores for each token pair using: $\alpha_{ij}=Softmax\left(\frac{Q_{i.}K_{j}^{T}}{\sqrt{Q_{i}}}\right)$ Compute attended features for each token: $A_{i}=\sum_{j=1}^{n}\alpha_{ij}V\;j$6.Aggregate global features A = ${\{A}_{1},A_{2},\ldots A_{n}\}$.7.Step 3: Attention-Based Fusion Combine local and global features using a weighted sum: $F_{Hybrid}=\beta F_{FFNet}+(1-\beta )A$8.Validation:9.if $\left||\;F_{Hybrid}|\right|$ exceeds predefined threshold ∈ then10.Add $F_{Hybrid}\;to\;F_{Hybrid}$
11.Else12.Flag $X_{i}$ for manual review.13.end if14.end for15.return $F_{Hybrid}$


The feature extraction process begins with preprocessing the MRI in where the X element of R is capped to H × W × D, ensuring uniformity in size and normalization to eliminate variations introduced by different imaging conditions. The preprocessed image is then passed through EfficentNet, a convolutional neural network known for its ability to learn hierarchical features while addressing the vanishing gradient problem through residual connections. Each residual block in EfficentNet refines the feature representation by learning the residual mapping F(x)=H(x)−x, where H(x) represents the desired mapping and x is the input. This formulation encourages the network to learn incremental adjustments to the features, leading to efficient and robust learning. The output of EfficentNet, $EFNet\in R^{H\prime \times W\prime \times D\prime }$, represents a high-dimensional feature map encapsulating spatial and local details such as texture patterns and structural anomalies relevant to PD. While EfficentNet excels at capturing local features, it is limited in capturing global dependencies across the entire image. To address this, the feature map $F_{FFNet}$ is flattened into a sequence of tokens $T=\{T_{1},T_{2},\ldots ,T_{n}\}$, where each token $T_{i}$ corresponds to a spatial region of the image. These tokens are then passed through an attention mechanism to compute relationships among different spatial regions. The attention mechanism calculates attention scores $\alpha_{ij}$ using the scaled dot-product attention formula:

(5) $$\alpha_{ij}=Softmax(\frac{Q_{i}.K_{j}^{T}}{\sqrt{d_{k}}})$$

where Q, K and V are the query, key, and value matrices derived from the token embeddings, and $d_{k}$ is the dimensionality of the key vectors. This mechanism assigns higher attention weights to regions that are more relevant to the overall context of the image. The attended features $A_{i}$ for each token are then computed as:

(6) $$A_{i}=\sum_{j=1}^{n}\alpha_{ij}\;,\;V_{j}$$

This process enables the model to capture long-range dependencies and global context, which are essential for understanding the spatial relationships between different brain regions. To integrate the strengths of both local and global feature extraction, attention-based fusion is employed. The local features $F_{FFNet}$ and the global dependencies $A=\{A_{1},A_{2},\ldots ,A_{n}\}$ are combined through a weighted sum:

(7) $$F_{Hybrid}=\beta F_{FFNet}+(1-\beta )A$$

where β∈[0,1] is a learnable parameter that balances the contribution of local and global features. This fusion process ensures that the resulting feature representation. $F_{Hybrid}$ retains fine-grained spatial details while incorporating the broader contextual information necessary for comprehensive analysis. The significance of this method is, in how it surpasses the constraints of models. EfficentNet focuses on extracting features that play a role in detecting minor anomalies and structural intricacies. However, its deficiency in grasping the picture is tackled through the utilization of the attention mechanism. On the one hand, the attention mechanism offers a perspective of the image by grasping connections among spatial areas, yet it depends on EfficentNet localized features to establish its context. These elements work together to form a model that’s ideal, for handling the intricacies of MRI data by providing durability and precision while being easy to understand.

The process of extracting features for diagnosing PD from MRI images consists of three stages. The initial image depicts a model MRI input that simulates a grayscale scan and serves as the data for further analysis. The second image showcases a feature map generated by the EfficientNet model, which emphasizes the local characteristics identified by a channel within the EfficientNet intermediate layers. Areas, with intensity in the feature map indicate where the neural network is paying attention in the MRI scans for potential regions of interest. The third image displays an attention map generated through attention-based fusion, illustrating the connections and interdependencies among regions. This map illustrates how the neural network incorporates context by assigning significance (represented by brighter areas) to spatial regions that influence the diagnosis. These visualizations work together to show a picture of how features are extracted in a hybrid way by balancing detailed local analysis, with a broader global perspective.

### 3.4. Feature Learning

After completing feature extraction using EfficentNet and an attention-based fusion mechanism to obtain hybrid features $F_{Hybrid}$, the next step is feature learning using Gated Recurrent Units (GRUs). GRUs are particularly important in this context because they excel at capturing sequential dependencies and learning temporal patterns in data. By incorporating GRUs, the extracted features $F_{Hybrid}$, which encapsulate both local and global context, can be further refined to emphasize relevant sequential information inherent in MRI data. This is crucial for improving the model’s understanding of complex patterns associated with PD.

The GRU architecture consists of two gates: a reset gate and an update gate [33]. These gates control the flow of information through the network, enabling it to selectively retain or forget aspects of the input. For each time step $t_{i}$, the reset gate $r_{t}$ decides how much of the past information to forget, while the update gate $z_{t}$ determines the extent to which the current state updates the previous state. The hidden state $h_{t}$, representing the learned feature representation at each step, is computed as:

(8) $$h_{t}=\left(1-z_{t}\right)⊙h_{t}-1+z_{t}⊙h_{t}^{\prime },$$

(9) $$h_{t}^{\prime }=tanh\left(W\cdot \left(r_{t}⊙h_{t-1}\right)+U\cdot x_{t}\right)$$

where $x_{t}$ is the input at time t, and W, U are trainable weight matrices.

The process begins by feeding the extracted features $F_{Hybrid}$ into the GRU, treating each token or feature vector as a sequential input. The GRU processes these sequentially, learning both short-term and long-term dependencies in the data. By leveraging its gating mechanisms, the GRU effectively emphasizes the most informative features while minimizing noise, enhancing the overall feature representation. The output of the GRU is a refined feature set that encapsulates temporal and sequential information.

### 3.5. Final Diagnosis

This section discusses the final diagnosis of PD that is performed by using a Fully Connected Neural network (FCN), which is essential for leveraging the refined features obtained from the GRU to make accurate predictions. The FC neural network plays a vital role in mapping the high-dimensional feature space to the binary classification task of distinguishing between PD and non-PD cases. Its importance lies in its ability to integrate and weigh the learned features effectively, ensuring that the most relevant patterns contribute to the final decision. An FC network consists of multiple layers of neurons, each fully connected to the neurons in the preceding layer. Mathematically, for an input feature vector *F* ∈ $R^{n}$ (output from the GRU), The output of the first hidden layer is computed as:

(10) $$h_{1}=\alpha \;(W_{1}F+b_{1})$$

where $W_{1}\in R^{m\times n}$ is the wright matrix $b_{1}\in \;R^{m}$ is the bias vector and α is the activation function. It is ReLU(x) = max(0, x). This process is repeated across subsequent layers, with each layer transforming its input into a new representation. For output layer, which performs binary classification, the activation function is typically a sigmoid function:

(11) $$y=Sigmoid\left(W_{o}h_{L-1}+b_{o}\right)$$

where $W_{o}$ and $b_{o}$ are the weights and bias of the output layer and y ∈ (0, 1) represents the probability of the input being classified as PD. The final predication y is determined as

(12) $$y=\left\{\begin{array}{l} 1\;\;\;if\;(y>0.5)\\ 0\;\;if\;(y<0.5) \end{array}\right.$$

where *y* = 1 corresponds to PD and *y* = 0 corresponds to non-PD. The FC neural network is trained using a binary cross-entropy loss function, defined as:

(13) $$L=-\frac{1}{N}\sum_{i=1}^{N}\left[y_{i}log(y_{i})+(1+y_{i})log(1-y_{i})\right]$$

where *N* is the number of training samples, $y_{i}$ is the true label, and $y_{i}$ is the predicted probability for the $i^{th}$ sample. This loss function ensures the network learns to minimize the difference between predicted and actual labels. By fully connecting all neurons across layers, the FC neural network captures complex interdependencies among features, effectively combining the temporal information from GRU and distinguishing PD from non-PD with high precision. Its adaptability and mathematical robustness make it an excellent choice for the final classification task in this pipeline.

## 4. Experimental Results and Evaluation

This section discusses the experimental evaluation and comparative analysis of FCN-PD.

### 4.1. Baseline Method

Based on the datasets outlined in Table 3, we assess the effectiveness of the proposed model by conducting a comparative analysis against the baseline models listed below.

- Baseline 1: Erdaş et al. [34]: used 2D and 3D CNN using T1-weighted MRIs for the detection of Parkinson’s disease.
- Baseline 2: Desai et al. [35]: presented a deep learning-based model that utilized 3D brain MRI scans for the detection of Parkinson’s disease.
- Baseline 3: Islam et al. [36]: presented a method based on implementation of DenseNet169 and CNN for Parkinson’s disease detection.

### 4.2. Result

**Figure 3:**
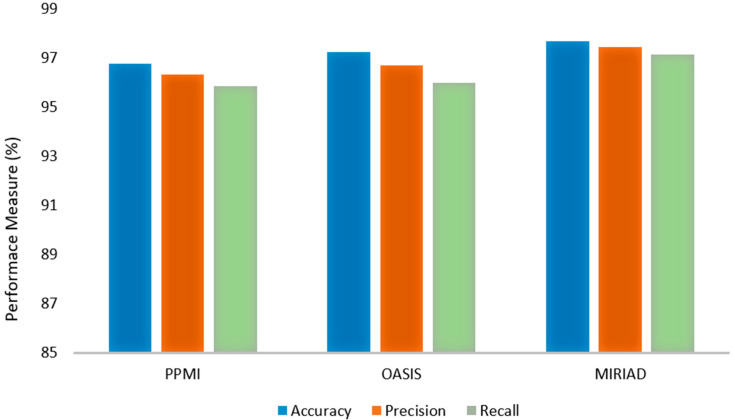
Experiments Results of FCN-PD.

The proposed FCN-PD framework demonstrated exceptional performance in diagnosing Parkinson’s disease (PD) across three publicly available MRI datasets: PPMI, OASIS, and MIRIAD. To ensure a robust evaluation, k-fold cross-validation was applied during the experiments, further validating the model’s reliability and generalization across different datasets. On the PPMI dataset, the model achieved an accuracy of 96.78%, with precision and recall scores of 96.34% and 95.84%, respectively. These results highlight the model’s ability to accurately classify PD and Healthy Control (HC) cases, minimizing false positives while effectively identifying true positives. The OASIS dataset yielded slightly higher performance metrics, with an accuracy of 97.23%, precision of 96.7%, and recall of 95.97%, demonstrating the model’s robustness in handling diverse MRI data characteristics. On the MIRIAD dataset, despite its smaller size, the model achieved its highest performance, with an accuracy of 97.67%, precision of 97.43%, and recall of 97.13%. The consistency of results across multiple datasets—validated through k-fold cross-validation—underscores FCN-PD’s ability to effectively integrate local and global feature representations through the combination of EfficientNet and the FCN. These findings confirm FCN-PD as a reliable and accurate tool for the early diagnosis of PD, with significant potential for integration into clinical workflows to enhance patient outcomes. Graphically the results are shown in Figure 3.

**Figure 4:**
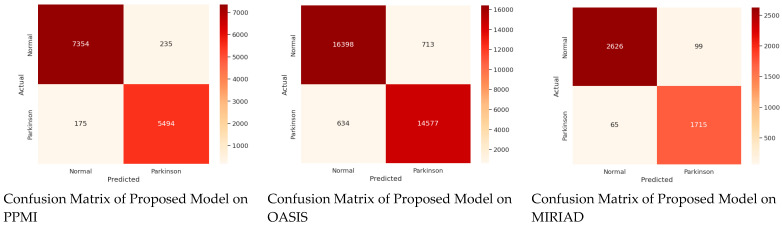
Performance measure based on confusion matrix of all three datasets.

The confusion matrices of the proposed FCN-PD model across the three datasets (PPMI, OASIS, and MIRIAD) demonstrate a high level of classification accuracy, effectively distinguishing between Parkinson’s disease (PD) and normal cases. The results are shown in Figure 4. In the PPMI dataset, the model accurately classifies a significant majority of cases, with a high number of true positives and true negatives, and a minimal number of misclassifications. Similarly, in the OASIS dataset, the FCN-PD model achieves robust performance, correctly identifying most PD and normal cases with very few false positives and false negatives. On the MIRIAD dataset, despite its smaller size, the model continues to demonstrate reliable diagnostic accuracy, correctly classifying the majority of cases. These results underscore the robustness of the FCN-PD model in delivering consistent and reliable performance across diverse datasets, paving the way for its potential integration into clinical workflows for early and accurate Parkinson’s disease diagnosis.

**Figure 5:**
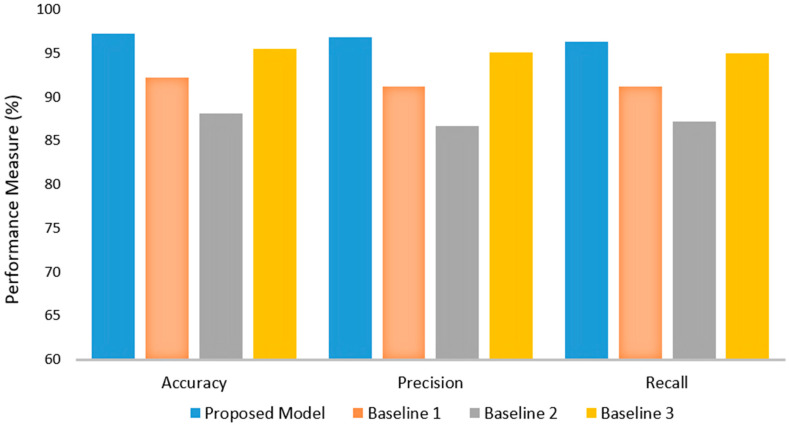
Comparative analysis of FCN-PD in terms of accuracy, precision, and recall.

The comparative analysis between the proposed FCN-PD model and three baseline methods demonstrates the superior performance of the proposed approach. Baseline 1, which employed 2D and 3D CNNs using T1-weighted MRIs, achieved an accuracy of 92.23%, precision of 91.18%, and recall of 91.21%. While it performed moderately well, its metrics were lower compared to the proposed model. Baseline 2, which utilized a deep learning-based model with 3D brain MRI scans, showed the lowest performance among the baselines, with an accuracy of 88.15%, precision of 86.62%, and recall of 87.2%. Baseline 3, based on DenseNet169 and CNN, achieved a higher accuracy of 95.49%, precision of 95.05%, and recall of 94.94%, indicating a robust approach but still falling short of the proposed model. In contrast, the proposed FCN-PD framework significantly outperformed all baselines, achieving an accuracy of 97.22%, precision of 96.82%, and recall of 96.31%. These results highlight the effectiveness of FCN-PD’s hybrid feature extraction strategy, leveraging EfficentNet and the FCN for both local and global context integration. The proposed model’s higher accuracy, precision, and recall confirm its capability to deliver more reliable and consistent PD diagnosis compared to existing methods. The results are shown in Figure 5.

The comparative analysis of log loss values across the three datasets PPMI, OASIS, and MIRIAD demonstrates the superior performance of the proposed FCN-PD model in minimizing prediction uncertainty compared to the baseline methods, as shown in Table 4. For the PPMI dataset, FCN-PD achieved the lowest log loss of 0.315, significantly outperforming Baseline 1 (0.340), Baseline 2 (0.530), and Baseline 3 (0.380). Similarly, on the OASIS dataset, FCN-PD achieved a log loss of 0.318, showing a clear advantage over Baseline 1 (0.350), Baseline 2 (0.540), and Baseline 3 (0.405). On the MIRIAD dataset, FCN-PD once again demonstrated the lowest log loss of 0.320, compared to Baseline 1 (0.355), Baseline 2 (0.515), and Baseline 3 (0.398). The consistent reduction in log loss across all datasets underscores the proposed model’s robustness in producing well-calibrated probability estimates, reflecting its ability to make confident predictions with minimized uncertainty. This improvement can be attributed to FCN-PD’s hybrid architecture, which integrates local and global feature representations, enabling more precise and reliable diagnostic performance compared to traditional models.

### 4.3. Ablation Study

Table 5 shows the results of the ablation study that clearly demonstrate the importance of each component in the proposed FCN-PD framework. When both U-Net and Auto Encoders are excluded during preprocessing, the overall performance drops significantly, with an average accuracy of only 79.8%, highlighting the critical role of preprocessing in preparing MRI data for analysis. The inclusion of U-Net, which segments essential brain regions like the substantia nigra, increases the average accuracy to 84.3%. Similarly, the Auto Encoder’s role in reducing noise and enhancing essential structural details is evident, as its exclusion results in a lower accuracy of 85.6%. This indicates that precise data preparation is fundamental to the framework’s success.

Further analysis reveals the significance of attention mechanisms in modeling global dependencies. Without these mechanisms, the average accuracy drops to 87.4%, underscoring their importance in capturing the contextual relationships within brain structures. EfficentNet, responsible for extracting localized spatial features such as cortical thickness and texture variations, also proves vital. When replaced with a simpler feature extractor, the framework’s accuracy reduces to 86.3%, confirming the necessity of advanced spatial feature extraction for robust diagnosis.

The FCN, which integrates local and global features using a hierarchical classification approach, is another crucial component. When replaced with a basic classifier, the average accuracy drops to 88.6%. This highlights the FCN’s ability to handle high-resolution MRI data efficiently and enhance feature representations. The full FCN-PD framework, with all components integrated, achieves the highest accuracy of 91.6%, proving the combined strength of the preprocessing, feature extraction, attention mechanisms, and hierarchical classification. These results validate the systematic design of the FCN-PD framework and its effectiveness in delivering precise and reliable PD diagnosis across diverse datasets, including PPMI, OASIS, and MIRAID.

## 5. Conclusions and Future Work Direction

This paper presents FCN-PD, an advanced deep learning framework for the diagnosis of PD using MRI data. The proposed model addresses the limitations of traditional diagnostic methods and existing computer-based approaches by integrating robust preprocessing, hybrid feature extraction, and efficient classification. U-Net ensures precise brain region segmentation, while Auto Encoders effectively denoise and enhance the MRI data. EfficentNet captures detailed local spatial features, and attention mechanisms model global dependencies, creating a comprehensive feature representation. The FCN further refines these features through hierarchical attention mechanisms, enabling accurate and efficient classification. Extensive evaluations on three benchmark MRI datasets demonstrate that FCN-PD outperforms traditional CNN-based and transformer-based methods, achieving state-of-the-art accuracy and robustness across different datasets. By leveraging advanced deep learning techniques and a well-designed pipeline, FCN-PD delivers reliable diagnostic results, paving the way for its integration into clinical workflows. This work not only advances the field of AI-driven medical imaging but also holds significant potential for improving early detection and management of PD, ultimately contributing to better patient outcomes and healthcare efficiency. Future work may explore real-time implementation and extend the framework to other neurodegenerative disorders.

## Figures and Tables

**Table 1:** The symptoms of PD at different stages.

Stage	Symptoms
Stage 1 (Mild)	- Tremors or shaking on one side of the body. - Slight rigidity or stiffness in muscles. - Decreased sense of smell (anosmia). - Minor difficulty with facial expressions and speech.
Stage 2 (Moderate)	- Symptoms become bilateral (both sides of the body). - Tremors, rigidity, and bradykinesia (slowness of movement) are more noticeable. - Difficulty with walking, posture, and balance. - Reduced coordination and fine motor control, affecting handwriting (micrographia).
Stage 3 (Mid-Stage)	- Noticeable balance problems and frequent falls. - Slowness of movement significantly impacts daily activities. - Difficulty with self-care tasks such as dressing and eating. - Increased rigidity and tremors.
Stage 4 (Advanced)	- Severe motor symptoms. - Requires assistance with daily activities (e.g., bathing, dressing, eating). - Limited ability to stand or walk without support. - Tremors and rigidity may be disabling.
Stage 5 (Late/Severe)	- Total dependence on caregivers for all activities. - Cannot stand or walk without assistance (may be confined to a wheelchair or bed). - Severe cognitive decline or dementia may occur. - Difficulty with swallowing, talking, and severe muscle stiffness.

**Table 3:** Dataset description.

Dataset Name	Dataset Source	Classes	Number of Images per Class
Parkinson’s Progression Marking Project (PPMI)	PPMI	1. Parkinson’s Disease (PD) 2. Healthy Control (HC)	PD: Over 1500 MRI images HC: Over 1000 MRI images
OASIS-3 (Open Access Series of Imaging Studies)	OASIS	1. Parkinson’s Disease (PD) 2. Healthy Control (HC)	PD: Not specifically focused on PD (approx. 200 PD-related scans available) HC: 1000 MRI scans
MIRIAD (MRI and Dementia Dataset)	MIRIAD	1. Parkinson’s Disease (PD) 2. Healthy Control (HC)	PD: 32 MRI scans HC: 31 MRI scans

**Table 4:** Log loss comparison of the proposed model with baselines.

Dataset	Baseline 1	Baseline 2	Baseline 3	FCN-PD (Proposed Model)
PPMI	0.340	0.530	0.380	0.325
OASIS	0.350	0.540	0.405	0.335
MIRIAD	0.355	0.515	0.398	0.330

**Table 5:** The component contribution wise evaluation of FCN-PD.

Configuration	Accuracy (%)	F1-Score (%)
Without Preprocessing (No U-Net, AutoEnc.)	79.8	78.5
Without U-Net (Segmentation Removed)	84.3	83.0
Without Auto Encoder (Noise Retention)	85.6	84.5
Without Attention Mechanisms	87.4	86.2
Without EfficentNet (Basic Feature Extraction)	86.3	85.4
Without FCN (Basic Classifier)	88.6	87.5
Full Pipeline (FCN-PD)	96.63	96.1

## Data Availability

The original contributions presented in this study are included in the article; further inquiries can be directed to the corresponding authors.
